# Analysis of a hyperbolic geometric model for visual texture perception

**DOI:** 10.1186/2190-8567-1-4

**Published:** 2011-06-06

**Authors:** Gregory Faye, Pascal Chossat, Olivier Faugeras

**Affiliations:** 1 NeuroMathComp Laboratory, INRIA, Sophia Antipolis, CNRS, ENS Paris, France; 2 Dept. of Mathematics, University of Nice Sophia-Antipolis, JAD Laboratory and CNRS, Parc Valrose, 06108 Nice Cedex 02, France

**Keywords:** Neural fields, nonlinear integro-differential equations, functional analysis, non-Euclidean analysis, stability analysis, hyperbolic geometry, hypergeometric functions, bumps

## Abstract

We study the neural field equations introduced by Chossat and Faugeras to model the representation and the processing of image edges and textures in the hypercolumns of the cortical area V1. The key entity, the structure tensor, intrinsically lives in a non-Euclidean, in effect hyperbolic, space. Its spatio-temporal behaviour is governed by nonlinear integro-differential equations defined on the Poincaré disc model of the two-dimensional hyperbolic space. Using methods from the theory of functional analysis we show the existence and uniqueness of a solution of these equations. In the case of stationary, i.e. time independent, solutions we perform a stability analysis which yields important results on their behavior. We also present an original study, based on non-Euclidean, hyperbolic, analysis, of a spatially localised bump solution in a limiting case. We illustrate our theoretical results with numerical simulations.

**AMS subject classifications: **30F45, 33C05, 34A12, 34D20, 34D23, 34G20, 37M05, 43A85, 44A35, 45G10, 51M10, 92B20, 92C20.

## 1 Introduction

The selectivity of the responses of individual neurons to external features is often the basis of neuronal representations of the external world. For example, neurons in the primary visual cortex (V1) respond preferentially to visual stimuli that have a specific orientation [1-3], spatial frequency [4], velocity and direction of motion [5], color [6]. A local network in the primary visual cortex, roughly 1 mm^2 ^of cortical surface, is assumed to consist of subgroups of inhibitory and excitatory neurons each of which is tuned to a particular feature of an external stimulus. These subgroups are the so-called Hubel and Wiesel hypercolumns of V1. We have introduced in [7] a new approach to model the processing of image edges and textures in the hypercolumns of area V1 that is based on a nonlinear representation of the image first order derivatives called the structure tensor [8,9]. We suggested that this structure tensor was represented by neuronal populations in the hypercolumns of V1. We also suggested that the time evolution of this representation was governed by equations similar to those proposed by Wilson and Cowan [10]. The question of whether some populations of neurons in V1 can represent the structure tensor is discussed in [7] but cannot be answered in a definite manner. Nevertheless, we hope that the predictions of the theory we are developing will help deciding on this issue.

Our present investigations were motivated by the work of Bressloff, Cowan, Golubitsky, Thomas and Wiener [11,12] on the spontaneous occurence of hallucinatory patterns under the influence of psychotropic drugs, and its extension to the structure tensor model. A further motivation was the following studies of Bressloff and Cowan [13,14,4] where they study a spatial extension of the ring model of orientation of Ben-Yishai [1] and Hansel, Sompolinsky [2]. To achieve this goal, we first have to better understand the local model, that is the model of a "texture" hypercolumn isolated from its neighbours.

The aim of this paper is to present a rigorous mathematical framework for the modeling of the representation of the structure tensor by neuronal populations in V1. We would also like to point out that the mathematical analysis we are developing here, is general and could be applied to other integro-differential equations defined on the set of structure tensors, so that even if the structure tensor were found to be not represented in a hypercolumn of V1, our framework would still be relevant. We then concentrate on the occurence of localized states, also called bumps. This is in contrast to the work of [7] and [15] where "spatially" periodic solutions were considered. The structure of this paper is as follows. In section 2 we introduce the structure tensor model and the corresponding equations. We also link our model to the ring model of orientations. In section 3 we use classical tools of evolution equations in functional spaces to analyse the problem of the existence and uniqueness of the solutions of our equations. In section 4 we study stationary solutions which are very important for the dynamics of the equation by analysing a nonlinear convolution operator and making use of the Haar measure of our feature space. In section 5, we push further the study of stationary solutions in a special case and we present a technical analysis involving hypergeometric functions of what we call a hyperbolic radially symmetric stationary-pulse in the high gain limit. Finally, in section 6, we present some numerical simulations of the solutions to verify the findings of the theoretical results.

## 2 The model

By definition, the structure tensor is based on the spatial derivatives of an image in a small area that can be thought of as part of a receptive field. These spatial derivatives are then summed nonlinearly over the receptive field. Let *I*(*x*, *y*) denote the original image intensity function, where *x *and *y *are two spatial coordinates. Let  denote the scale-space representation of *I *obtained by convolution with the Gaussian kernel :

The gradient  is a two-dimensional vector of coordinates ,  which emphasizes image edges. One then forms the 2 × 2 symmetric matrix of rank one , where **^T ^**indicates the transpose of a vector. The set of 2 × 2 symmetric positive semidefinite matrices of rank one will be noted S^+^(1, 2) throughout the paper (see [16] for a complete study of the set S^+^(*p*, *n*) of *n *× *n *symmetric positive semidefinite matrices of fixed-rank *p *<*n*). By convolving  componentwise with a Gaussian  we finally form the tensor structure as the symmetric matrix:

where we have set for example:

Since the computation of derivatives usually involves a stage of scale-space smoothing, the definition of the structure tensor requires two scale parameters. The first one, defined by *σ*_1_, is a local scale for smoothing prior to the computation of image derivatives. The structure tensor is insensitive to noise and details at scales smaller than *σ*_1_. The second one, defined by *σ*_2_, is an integration scale for accumulating the nonlinear operations on the derivatives into an integrated image descriptor. It is related to the characteristic size of the texture to be represented, and to the size of the receptive fields of the neurons that may represent the structure tensor.

By construction,  is symmetric and non negative as  by the inequality of Cauchy-Schwarz, then it has two orthonormal eigenvectors e_1_, e_2 _and two non negative corresponding eigenvalues λ_1 _and λ_2 _which we can always assume to be such that λ_1 _≥ λ_2 _≥ 0. Furthermore the spatial averaging distributes the information of the image over a neighborhood, and therefore the two eigenvalues are always positive. Thus, the set of the structure tensors lives in the set of 2 × 2 symmetric positive definite matrices, noted SPD(2, ℝ) throughout the paper. The distribution of these eigenvalues in the (λ_1_, λ_2_) plane reflects the local organization of the image intensity variations. Indeed, each structure tensor can be written as the linear combination:(1)

where **I**_2 _is the identity matrix and . Some easy interpretations can be made for simple examples: constant areas are characterized by λ_1 _= λ_2 _≈ 0, straight edges are such that λ_1 _≫ λ_2 _≈ 0, their orientation being that of **e**_2_, corners yield λ_1 _≥ λ_2 _≫ 0. The coherency *c *of the local image is measured by the ratio , large coherency reveals anisotropy in the texture.

We assume that a hypercolumn of V1 can represent the structure tensor in the receptive field of its neurons as the average membrane potential values of some of its membrane populations. Let  be a structure tensor. The time evolution of the average potential  for a given column is governed by the following neural mass equation adapted from [7] where we allow the connectivity function *W *to depend upon the time variable *t *and we integrate over the set of 2 × 2 symmetric definite-positive matrices:(2)

The nonlinearity *S *is a sigmoidal function which may be expressed as:

where *μ *describes the stiffness of the sigmoid. *I*_ext _is an external input.

The set SPD(2) can be seen as a foliated manifold by way of the set of special symmetric positive definite matrices SSPD(2) = SPD(2)∩SL(2, ℝ). Indeed, we have: . Furthermore,  where  is the Poincaré Disk, see e.g. [7]. As a consequence we use the following foliation of , which allows us to write for all ,  with . , *z *and Δ are related by the relation  and the fact that *z *is the representation in  of  with .

It is well-known [17] that  (and hence SSPD(2)) is a two-dimensional Riemannian space of constant sectional curvature equal to -1 for the distance noted *d*_2 _defined by

The isometries of , that are the transformations that preserve the distance *d*_2 _are the elements of unitary group U(1, 1). In appendix A we describe the basic structure of this group. It follows, e.g. [18,7], that SDP(2) is a three-dimensional Riemannian space of constant sectional curvature equal to -1 for the distance noted *d*_0 _defined by

As shown in proposition B.0.1 of appendix B it is possible to express the volume element  in (*z*_1_, *z*_2_, Δ) coordinates with *z *= *z*_1 _+ *iz*_2_:

We note  and equation (2) can be written in (*z*, Δ) coordinates:

We get rid of the constant  by redefining *W *as .(3)

In [7], we have assumed that the representation of the local image orientations and textures is richer than, and contains, the local image orientations model which is conceptually equivalent to the direction of the local image intensity gradient. The richness of the structure tensor model has been expounded in [7]. The embedding of the ring model of orientation in the structure tensor model can be explained by the intrinsic relation that exists between the two sets of matrices SPD(2, ℝ) and S^+^(1, 2). First of all, when *σ*_2 _goes to zero, that is when the characteristic size of the structure becomes very small, we have , which means that the tensor  degenerates to a tensor , which can be interpreted as the loss of one dimension. We can write each  as , where *u *= (cos *θ*, sin *θ*)**^T ^**and (*r*, *θ*) is the polar representation of *x*. Since, *x *and -*x *correspond to the same , *θ *is equated to *θ *+ *kπ*, . Thus , where ℙ^1 ^is the real projective space of dimension 1 (lines of ℝ^2^). Then the integration scale *σ*_2_, at which the averages of the estimates of the image derivatives are computed, is the link between the classical representation of the local image orientations by the gradient and the representation of the local image textures by the structure tensor. It is also possible to highlight this explanation by coming back to the interpretation of straight edges of the previous paragraph. When λ_1 _≫ λ_2 _≈ 0 then  and the orientation is that of **e**_2_. We denote by ℙ the projection of a 2 × 2 symmetric definite positive matrix on the set S^+^(1, 2) defined by:

where  is as in equation (1). We can introduce a metric on the set S^+^(1, 2) which is derived from a well-chosen Riemannian quotient geometry (see [16]). The resulting Riemannian space has strong geometrical properties: it is geodesically complete and the metric is invariant with respect to all transformations that preserve angles (orthogonal transformations, scalings and pseudoinversions). Related to the decomposition , a metric on the space S^+^(1, 2) is given by:

The space S^+^(1, 2) endowed with this metric is a Riemannian manifold (see [16]). Finally, the distance associated to this metric is given by:

where  and (*r_i_*, *θ_i_*) denotes the polar coordinates of *x_i _*for *i *= 1, 2. The volume element in (*r*, *θ*) coordinates is:

where we normalize to 1 the volume element for the *θ *coordinate.

Let now  be a symmetric positive semidefinite matrix. The average potential *V*(*τ*, *t*) of the column has its time evolution that is governed by the following neural mass equation which is just a projection of equation (2) on the subspace S^+^(1, 2):(4)

In (*r*, *θ*) coordinates, (4) is rewritten as:

This equation is richer than the ring model of orientation as it contains an additional information on the contrast of the image in the orthogonal direction of the prefered orientation. If one wants to recover the ring model of orientation tuning in the visual cortex as it has been presented and studied by [1,2,19], it is sufficient i) to assume that the connectivity function is time-independent and has a convolutional form:

and ii) to look at semi-homogeneous solutions of equation 4, i.e., solutions which do not depend upon the variable *r*. We finally obtain:(5)

where:

It follows from the above discussion that the structure tensor contains, at a given scale, more information than the local image intensity gradient at the same scale and that it is possible to recover the ring model of orientations from the structure tensor model.

The aim of the following sections is to establish that (3) is well-defined and to give necessary and sufficient conditions on the different parameters in order to prove some results on the existence and uniqueness of a solution of (3).

## 3 The existence and uniqueness of a solution

In this section we provide theoretical and general results of existence and uniqueness of a solution of (2). In the first subsection 3.1 we study the simpler case of the homogeneous solutions of (2), i.e. of the solutions that are independent of the tensor variable . This simplified model allows us to introduce some notations for the general case and to establish the useful lemma 3.1.1. We then prove in 3.2 the main result of this section, that is the existence and uniqueness of a solution of (2). Finally we develop the useful case of the semi-homogeneous solutions of (2), i.e. of solutions that depend on the tensor variable but only through its *z *coordinate in .

### 3.1 Homogeneous solutions

A homogeneous solution to (2) is a solution *V *that does not depend upon the tensor variable  for a given homogenous input *I*(*t*) and a constant initial condition *V*_0_. In (*z*, Δ) coordinates, a homogeneous solution of (3) is defined by:

where:(6)

Hence necessary conditions for the existence of a homogeneous solution are that:

• the double integral (6) is convergent,

•  does not depend upon the variable (*z*, Δ). In that case, we write  instead of .

In the special case where *W*(*z*, Δ, *z*', Δ', *t*) is a function of only the distance *d*_0 _between (*z*, Δ) and (*z*', Δ'):

the second condition is automatically satisfied. The proof of this fact is given in lemma D.0.2 of appendix D. To summarize, the homogeneous solutions satisfy the differential equation:(7)

#### 3.1.1 A first existence and uniqueness result

Equation (3) defines a Cauchy's problem and we have the following theorem.

**Theorem 3.1.1**. *If the external input I_ext_(t) and the connectivity function **are continuous on some closed interval J containing *0*, then for all V*_0 _*in *ℝ, *there exists a unique solution of *(7) *defined on a subinterval J*_0 _*of J containing *0 *such that V *(0) *= V*_0_.

*Proof*. It is a direct application of Cauchy's theorem on differential equations. We consider the mapping *f *: *J *× ℝ → ℝ defined by:

It is clear that *f *is continuous from *J *× ℝ to ℝ. We have for all *x*, *y *∈ ℝ and *t *∈ *J*:

where .

Since,  is continuous on the compact interval *J*, it is bounded there by *C *> 0 and:

□

We can extend this result to the whole time real line if *I *and  are continuous on ℝ.

**Proposition 3.1.1**. *If I_ext _and **are continuous on *ℝ^+^*, then for all V*_0 _*in *ℝ*, there exists a unique solution of (7) defined on *ℝ^+ ^*such that V *(0) *= V*_0_.

*Proof*. We have already shown the following inequality:

Then *f *is locally Lipschitz with respect to its second argument. Let *V *be a maximal solution on *J*_0 _and we denote by *β *the upper bound of *J*_0_. We suppose that *β *< + ∞. Then we have for all *t *≥ 0:

where *S^m ^*= sup_*x*∈ℝ _|*S*(*x*)|.

This implies that the maximal solution *V *is bounded for all *t *∈ [0, *β*], but theorem C.0.2 of appendix C ensures that it is impossible. Then, it follows that necessarily *β *= + ∞.   □

#### 3.1.2 Simplification of (6) in a special case

##### Invariance

In the previous section, we have stated that in the special case where *W *was a function of the distance between two points in , then  did not depend upon the variables (*z*, Δ). As already said in the previous section, the following result holds (see proof of lemma D.0.2 of appendix D).

**Lemma 3.1.1**. *Suppose that W is a function of d*_0 _* only. Then **does not depend upon the variable *.

##### Mexican hat connectivity

In this paragraph, we push further the computation of  in the special case where *W *does not depend upon the time variable *t *and takes the special form suggested by Amari in [20], commonly referred to as the "Mexican hat" connectivity. It features center excitation and surround inhibition which is an effective model for a mixed population of interacting inhibitory and excitatory neurons with typical cortical connections. It is also only a function of *d*_0 _.

In detail, we have:

where:

with 0 ≤ *σ*_1 _≤ *σ*_2 _and 0 ≤ A ≤ 1.

In this case we can obtain a very simple closed-form formula for  as shown in the following lemma.

**Lemma 3.1.2**. *When W is the specific Mexican hat function just defined then*:(8)

*where **erf **is the error function defined as*:

*Proof*. The proof is given in lemma E.0.3 of appendix E.   □

### 3.2 General solution

We now present the main result of this section about the existence and uniqueness of solutions of equation (2). We first introduce some hypotheses on the connectivity function *W*. We present them in two ways: first on the set of structure tensors considered as the set SPD(2), then on the set of tensors seen as . Let *J *be a subinterval of ℝ. We assume that:

• ,

•  where **W **is defined as  for all  where Id_2 _is the identity matrix of ℳ_2_(ℝ),

•  where .

Equivalently, we can express these hypotheses in (*z*, Δ) coordinates:

• ,

•  where **W **is defined as **W**(*z*, Δ, *t*) = *W *(*d*_2_(*z*, 0), | log(Δ)|, *t*) for all ,

•  where

#### 3.2.1 Functional space setting

We introduce the following mapping *f^g ^*: (*t*, *ϕ*) → *f^g^*(*t*, *ϕ*) such that:(9)

Our aim is to find a functional space  where (3) is well-defined and the function *f^g ^*maps  to  for all *t*s. A natural choice would be to choose *ϕ *as a -integrable function of the space variable with 1 ≤ *p *< +∞. Unfortunately, the homogeneous solutions (constant with respect to (*z*, Δ)) do not belong to that space. Moreover, a valid model of neural networks should only produce bounded membrane potentials. That is why we focus our choice on the functional space . As  is an open set of ℝ^3^,  is a Banach space for the norm:.

**Proposition 3.2.1**. *If  with  and W satisfies hypotheses (**H1**bis)-(**H3**bis) then f ^g ^is well-defined and is from J × ** to *.

*Proof*. , we have:

   □

#### 3.2.2 The existence and uniqueness of a solution of (3)

We rewrite (3) as a Cauchy problem:(10)

**Theorem 3.2.1**. *If the external current I_ext _belongs to  with J an open interval containing *0 *and W satisfies hypotheses (**H1**bis)-(**H3**bis), then for all V*_0 _*∈ **, there exists a unique solution of (10) defined on a subinterval J*_0 _*of J containing *0 *such that V (z*, Δ, 0*) = V*_0_*(z*, Δ*) for all *.

*Proof*. We prove that *f^g ^*is continuous on *J *× . We have

and therefore

Because of condition (**H2**) we can choose |*t *-*s*| small enough so that  is arbitrarily small. This proves the continuity of *f^g^*. Moreover it follows from the previous inequality that:

with . This ensures the Lipschitz continuity of *f^g ^*with respect to its second argument, uniformly with respect to the first. The Cauchy-Lipschitz theorem on a Banach space yields the conclusion.   □

**Remark 3.2.1**. *Our result is quite similar to those obtained by Potthast and Graben in *[21]. *The main differences are that, first, we allow the connectivity function to depend upon the time variable t and, second, that our space features is no longer a *ℝ*^n ^but a Riemanian manifold. In their article, Potthast and Graben also work with a different functional space by assuming more regularity for the connectivity function W and then obtain more regularity for their solutions*.

**Proposition 3.2.2**. *If the external current I_ext _belongs to  and W satisfies hypotheses (**H1**bis)-(**H3**bis) with J = *ℝ*^+^, then for all V*_0 _*∈ **, there exists a unique solution of (10) defined on *ℝ*^+ ^such that V (z*, Δ, 0*) = V*_0_*(z*, Δ*) for all *.

*Proof*. We have just seen in the previous proof that *f^g ^*is globally Lipschitz with respect to its second argument:

then theorem C.0.3 of the appendix C gives the conclusion.   □

#### 3.2.3 The intrinsic boundedness of a solution of (3)

In the same way as in the homogeneous case, we show a result on the boundedness of a solution of (3).

**Proposition 3.2.3**. *If the external current I_ext _belongs to **and is bounded in time  and W satisfies hypotheses (**H1**bis)-(**H3**bis) with J = *ℝ*^+^, then the solution of (10) is bounded for each initial condition V*_0 _*∈ *.

Let us set:

where .

*Proof*. Let *V *be a solution defined on ℝ^+^. Then we have for all *t *∈ ℝ^+*^:

The following upperbound holds(11)

We can rewrite (11) as:(12)

If  this implies  for all *t *> 0 and hence ||*V*(*t*)||_ℱ _<*ρ^g ^*for all *t *> 0, proving that *B_p _*is stable. Now assume that ||*V*(*t*)||_ℱ _<*ρ^g ^*for all *t *≥ 0. The inequality (12) shows that for *t *large enough this yields a contradiction. Therefore there exists *t*_0 _> 0 such that ||*V*(*t*_0_)||_ℱ _<*ρ^g^*. At this time instant we have

and hence

   □

The following corollary is a consequence of the previous proposition.

**Corollary 3.2.1**. *If ** and ** then:*

### 3.3 Semi-homogeneous solutions

A semi-homogeneous solution of (3) is defined as a solution which does not depend upon the variable Δ. In other words, the populations of neurons is not sensitive to the determinant of the structure tensor, that is to the contrast of the image intensity. The neural mass equation is then equivalent to the neural mass equation for tensors of unit determinant. We point out that semi-homogeneous solutions were previously introduced in [7] where a bifurcation analysis of what they called H-planforms was performed. In this section, we define the framework in which their equations make sense without giving any proofs of our results as it is a direct consequence of those proven in the general case. We rewrite equation (3) in the case of semi-homogeneous solutions:(13)

where

We have implicitly made the assumption, that *W^sh ^*does not depend on the coordinate Δ. Some conditions under which this assumption is satisfied are described below and are the direct transductions of those of the general case in the context of semi-homogeneous solutions.

Let *J *be an open interval of ℝ. We assume that:

• ,

•  where **W***^sh ^*is defined as **W***^sh ^*(*z*, *t*) = *w^sh^*(*d*_2_(*z*, 0), *t*) for all ,

•  where .

Note that conditions (**C1**)-(**C2**) and lemma 3.1.1 imply that for all , . And then, for all , the mapping *z*' → *W^sh^*(*z*, *z'*, *t*) is integrable on .

From now on,  and the Fischer-Riesz's theorem ensures that  is a Banach space for the norm: .

**Theorem 3.3.1**. *If the external current I_ext _belongs to **with J an open interval containing *0 *and W^sh ^satisfies conditions (**C1**)-(**C3**), then for all V*_0 _*∈ **, there exists a unique solution of (13) defined on a subinterval J*_0 _*of J containing *0.

This solution, defined on the subinterval *J *of ℝ can in fact be extended to the whole real line, and we have the following proposition.

**Proposition 3.3.1**. *If the external current I_ext _belongs to **and W^sh ^satisfies conditions (**C1**)-(**C3**) with J = *ℝ^+^*, then for all V*_0 _*∈ **, there exists a unique solution of (13) defined on *ℝ^+^.

We can also state a result on the boundedness of a solution of (13):

**Proposition 3.3.2**. *Let *, *with *. *The open ball B_p _of ** of center *0 *and radius p is stable under the dynamics of equation (13)*. *Moreover it is an attracting set for this dynamics and if V*_0 _∉ *B_ρ _and T *= inf{*t *> 0 *such that V*(*t*) ∈ *B_P_*} *then:*

## 4 Stationary solutions

We look at the equilibrium states, noted  of (3), when the external input *I *and the connectivity W do not depend upon the time. We assume that *W *satisfies hypotheses (**H1**bis)-(**H2**bis). We redefine for convenience the sigmoidal function to be:

so that a stationary solution (independent of time) satisfies:(14)

We define the nonlinear operator from  to , noted , by:(15)

Finally, (14) is equivalent to:

### **4.1 Study of the nonlinear operator **

We recall that we have set for the Banach space  and proposition 3.2.1 shows that . We have the further properties:

**Proposition 4.1.1**. *satisfies the following properties*:

• ,

• *is continuous on *ℝ^+^,

*Proof*. The first property was shown to be true in the proof of theorem 3.3.1. The second property follows from the following inequality:

□

We denote by  and  the two operators from  to  defined as follows for all *V *∈  and all :(16)

and

where *H *is the Heaviside function.

It is straightforward to show that both operators are well-defined on  and map  to . Moreover the following proposition holds.

**Proposition 4.1.2**. *We have*

*Proof*. It is a direct application of the dominated convergence theorem using the fact that:

   □

### **4.2 The convolution form of the operator ****in the semi-homogeneous case**

It is convenient to consider the functional space  to discuss semi-homogeneous solutions. A semi-homogeneous persistent state of (3) is deduced from (14) and satisfies:(17)

where the nonlinear operator  from *^sh ^*to *^sh ^*is defined for all V ∈ *^sh^*and ; by:

We define the associated operators, , :

We rewrite the operator  in a convenient form by using the convolution in the hyperbolic disk. First, we define the convolution in a such space. Let *O *denote the center of the Poincaré disk that is the point represented by *z *= 0 and *dg *denote the Haar measure on the group *G *= *SU*(1, 1) (see [22] and appendix A), normalized by:

for all functions of . Given two functions *f*_1_, *f*_2 _in  we define the convolution * by:

We recall the notation .

**Proposition 4.2.1**. *For all μ *≥ 0 *and V *∈ *^sh ^we have*:(18)

*Proof*. We only prove the result for. Let , then:

and for all *g *∈ *SU*(1, 1), *d*_2_(*z*, *z'*) = *d*_2_(*g*·*z*, *g*·*z'*) so that:

   □

Let *b *be a point on the circle . For , we define the "inner product" <*z*, *b *> to be the algebraic distance to the origin of the (unique) horocycle based at *b *through *z *(see [7]). Note that <*z*, *b *> does not depend on the position of *z *on the horocycle. The Fourier transform in  is defined as (see [22]):

for a function  such that this integral is well-defined.

**Lemma 4.2.1**. *The Fourier transform in *, * of **W**^sh ^does not depend upon the variable . Proof*. For all *λ *∈ ℝ and ,

We recall that for all *ϕ *∈ ℝ *r_ϕ _*is the rotation of angle *ϕ *and we have **W***^sh^*(*r_ϕ _*·*z*) = **W***^sh^*(*z*), *dm*(*z*) = *dm*(*r_ϕ _*·*z*) and <*z*, *b *> = <*r_ϕ _*·*z*, *r_ϕ _*·*b *>, then:

   □

We now introduce two functions that enjoy some nice properties with respect to the Hyperbolic Fourier transform and are eigenfunctions of the linear operator .

**Proposition 4.2.2**. *Let e_λ_*, *_b_*(*z*) = *e*^(-*iλ*+1)<*z*, *b*>^*and ** then:*

• 

• 

*Proof*. We begin with  and use the horocyclic coordinates. We use the same changes of variables as in lemma 3.1.1:

By rotation, we obtain the property for all .

For the second property [[22], Lemma 4.7] shows that:

A consequence of this proposition is the following lemma.   □

**Lemma 4.2.2**. *The linear operator **is not compact and for all μ *≥ 0*, the nonlinear operator **is not compact*.

*Proof*. The previous proposition 4.2.2 shows that  has a continuous spectrum which iimplies that is not a compact operator.

Let *U *be in *^sh^*, for all *V *∈ *^sh ^*we differentiate  and compute its Frechet derivative:

If we assume further that *U *does not depend upon the space variable *z*, *U*(*z*) = *U*_0 _we obtain:

If  was a compact operator then its Frechet derivative  would also be a compact operator, but it is impossible. As a consequence,  is not a compact operator.   □

### **4.3 The convolution form of the operator ****in the general case**

We adapt the ideas presented in the previous section in order to deal with the general case. We recall that if *H *is the group of positive real numbers with multiplication as operation, then the Haar measure *dh *is given by . For two functions *f*_1_, *f*_2 _in  we define the convolution ⋆ by:

We recall that we have set by definition: **W**(*z*, Δ) = *W*(*d*_2_(*z*, 0), |log(Δ)|).

**Proposition 4.3.1**. *For all μ *≥ 0 *and V *∈ *we have:*(19)

*Proof*. Let (*z*, Δ) be in . We follow the same ideas as in proposition 4.2.1 and prove only the first result. We have

   □

We next assume further that the function **W **is separable in *z*, Δ and more precisely that **W**(*z*, Δ) = **W**_1_(*z*)**W**_2_(log(Δ)) where **W**_1_(*z*) = *W*_1_(*d*_2_(*z*, 0)) and **W**_2_(log(Δ)) = *W*_2_(|log(Δ)|) for all . The following proposition is an echo to proposition 4.2.2.

**Proposition 4.3.2**. *Let e_λ_*,*_b_*(*z*) = *e*^(-*iλ*+1)<*z*, *b*>^, * and h_ξ_*(Δ) = *e*^*iξ *log(Δ) ^*then:*

• 

• 

*Where ** is the usual Fourier transform of ***W**_2_.

*Proof*. The proof of this proposition is exactly the same as for proposition 4.2.2. Indeed:

   □

A straightforward consequence of this proposition is an extension of lemma 4.2.2 to the general case:

**Lemma 4.3.1**. *The linear operator **is not compact and for all μ *≥ 0*, the nonlinear operator **is not compact*.

### 4.4 The set of the solutions of (14)

Let  be the set of the solutions of (14) for a given slope parameter *μ*:

We have the following proposition.

**Proposition 4.4.1**. *If the input current I_ext _is equal to a constant **, i.e. does not depend upon the variables *(*z*, Δ) *then for all *, *. In the general case **, if the condition **is satisfied, then Card *.

*Proof*. Due to the properties of the sigmoid function, there always exists a constant solution in the case where *I*_ext _is constant. In the general case where , the statement is a direct application of the Banach fixed point theorem, as in [23].   □

**Remark 4.4.1**. *If the external input does not depend upon the variables *(*z*, Δ) *and if the condition **is satisfied, then there exists a unique stationary solution by application of proposition 4.4.1. Moreover, this stationary solution does not depend upon the variables *(*z*, Δ) *because there always exists one constant stationary solution when the external input does not depend upon the variables *(*z*, Δ)*. Indeed equation *(14) *is then equivalent to:*

*and β does not depend upon the variables *(*z*, Δ) *because of lemma 3.1.1. Because of the property of the sigmoid function S, the equation ** has always one solution*.

*If on the other hand the input current does depend upon these variables, is invariant under the action of a subgroup of U*(1, 1)*, the group of the isometries of **(see appendix A), and the condition **is satisfied, then the unique stationary solution will also be invariant under the action of the same subgroup. We refer the interested reader to our work *[15]* on equivariant bifurcation of hyperbolic planforms on the subject*.

*When the condition **is satisfied we call primary stationary solution the unique solution in *.

### 4.5 Stability of the primary stationary solution

In this subsection we show that the condition  guarantees the stability of the primary stationary solution to (3).

**Theorem 4.5.1**. *We suppose that ** and that the condition **is satisfied, then the associated primary stationary solution of *(3) *is asymtotically stable*.

*Proof*. Let  be the primary stationary solution of (3), as  is satisfied. Let also *V_μ _*be the unique solution of the same equation with some initial condition , see theorem 3.3.1. We introduce a new function  which satisfies:

where ,  and the vector Θ(*X*(*z*, Δ, *t*)) is given by  with . We note that, because of the definition of Θ and the mean value theorem |Θ(*X*(*z*, Δ, *t*))| ≤ *μ*|*X*(*z*, Δ, *t*)|. This implies that |Θ(*r*)| ≤ |*r*| for all *r *∈ ℝ.

If we set: *G*(*t*) = *e^αt^||X*(*t*)||_∞_, then we have:

and *G *is continuous for all *t *≥ 0. The Gronwall inequality implies that:

and the conclusion follows.   □

## 5 Spatially localised bumps in the high gain limit

In many models of working memory, transient stimuli are encoded by feature-selective persistent neural activity. Such stimuli are imagined to induce the formation of a spatially localised bump of persistent activity which coexists with a stable uniform state. As an example, Camperi and Wang [24] have proposed and studied a network model of visuo-spatial working memory in prefontal cortex adapted from the ring model of orientation of Ben-Yishai and colleagues [1]. Many studies have emerged in the past decades to analyse these localised bumps of activity [25-29], see the paper by Coombes for a review of the domain [30]. In [25,26,28], the authors have examined the existence and stability of bumps and multi-bumps solutions to an integro-differential equation describing neuronal activity along a single spatial domain. In [27,29] the study is focused on the two-dimensional model and a method is developed to approximate the integro-differential equation by a partial differential equation which makes possible the determination of the stability of circularly symmetric solutions. It is therefore natural to study the emergence of spatially localized bumps for the structure tensor model in a hypercolumn of V1. We only deal with the reduced case of equation (13) which means that the membrane activity does not depend upon the contrast of the image intensity, keeping the general case for future work.

In order to construct exact bump solutions and to compare our results to previous studies [25-29], we consider the high gain limit *μ *→ ∞ of the sigmoid function. As above we denote by *H *the Heaviside function defined by *H*(*x*) = 1 for *x *≥ 0 and *H*(*x*) = 0 otherwise. Equation (13) is rewritten as:(20)

We have introduced a threshold *κ *to shift the zero of the Heaviside function. We make the assumption that the system is spatially homogeneous that is, the external input *I *does not depend upon the variables *t *and the connectivity function depends only on the hyperbolic distance between two points of . For illustrative purposes, we will use the exponential weight distribution as a specific example throughout this section:(21)

The theoretical study of equation (20) has been done in [21] where the authors have imposed strong regularity assumptions on the kernel function W, such as Hölder continuity, and used compactness arguments and integral equation techniques to obtain a global existence result of solutions to (20). Our approach is very different, we follow that of [25,31,29] by proceeding in a constructive fashion. In a first part, we define what we call a hyperbolic radially symmetric bump and present some preliminary results for the linear stability analysis of the last part. The second part is devoted to the proof of a technical theorem 5.1.1 which is stated in the first part. The proof uses results on the Fourier transform introduced in section 4, hyperbolic geometry and hypergeometric functions. Our results will be illustrated in the following section 6.

### 5.1 Existence of hyperbolic radially symmetric bumps

From equation (20) a general stationary pulse satisfies the equation:

For convenience, we note *M*(*z*, *K*) the integral *∫_K _W*(*z*, *z*')dm(*z*') with . The relation *V *(*z*) = *κ *holds for all *z *∈ ∂*K*.

**Definition 5.1.1**. *V is called a hyperbolic radially symmetric stationary-pulse solution of *(20) *if V depends only upon the variable r and is such that:*

*and is a fixed point of equation *(20)*:*(22)

*where ** is a Gaussian input and ** is defined by the following equation:*

*and B_h_*(0, *ω*) *is a hyperbolic disk centered at the origin of hyperbolic radius ω*.

From symmetry arguments there exists a hyperbolic radially symmetric stationary-pulse solution *V*(*r*) of (20), furthermore the threshold *κ *and width *ω *are related according to the self-consistency condition(23)

where

**Figure 1:**
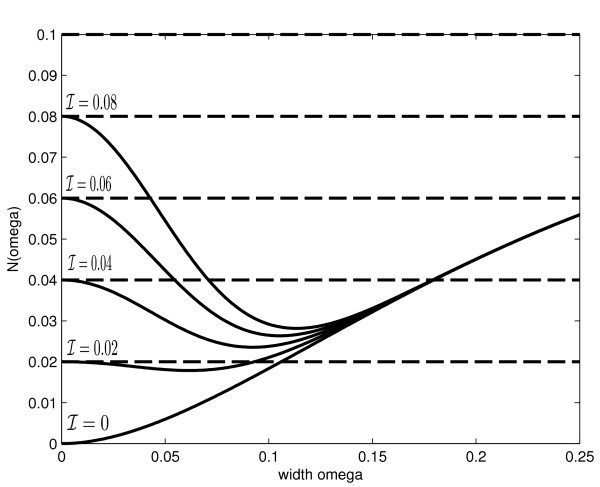
**Plot of *N*(*ω*) defined in (23) as a function of the pulse width *ω *for several values of the input amplitude  and for a fixed input width *σ *= 0.05**. The horizontal dashed lines indicate different values of *ακ*. The connectivity function is given in equation (21) and the parameter *b *is set to *b *= 0.2.

The existence of such a bump can then be established by finding solutions to (23) The function *N*(*ω*) is plotted in Figure 1 for a range of the input amplitude . The horizontal dashed lines indicate different values of *ακ*, the points of intersection determine the existence of stationary pulse solutions. Qualitatively, for sufficiently large input amplitude  we have *N*'(0) < 0 and it is possible to find only one solution branch for large *ακ*. For small input amplitudes  we have *N*'(0) > 0 and there always exists one solution branch for *αβ *<*γ_c _*≈ 0.06. For intermediate values of the input amplitude , as *αβ *varies, we have the possiblity of zero, one or two solutions. Anticipating the stability results of section 5.3, we obtain that when *N*'(*ω*) < 0 then the corresponding solution is stable.

We end this subsection with the usefull and technical following formula.

**Theorem 5.1.1**. *For all **:*(24)

*Where ** is the Fourier Helgason transform of ** and*

*with α *+ *β *+ 1 = *ρ and F is the hypergeometric function of first kind*.

**Remark 5.1.1**. *We recall that F admits the integral representation *[32]:

*with *ℜ(*γ*) > ℜ(*β*) > 0.

**Remark 5.1.2**. *In section 4 we introduced the function **. In *[22]*, it is shown that:*

**Remark 5.1.3**. *Let us point out that this result can be linked to the work of Folias and Bressloff in *[31]* and then used in *[29]*. They constructed a two-dimensional pulse for a general, radially symmetric synaptic weight function. They obtain a similar formal representation of the integral of the connectivity function w over the disk B*(*O*, *a*) *centered at the origin O and of radius a. Using their notations*,

*where J_ν_*(*x*) *is the Bessel function of the first kind and ** is the real Fourier transform of w. In our case, instead of the Bessel function, we find ** which is linked to the hypergeometric function of the first kind*.

We now show that for a general monotonically decreasing weight function *W*, the function  is necessarily a monotonically decreasing function of *r*. This will ensure that the hyperbolic radially symmetric stationary-pulse solution (22) is also a monotonically decreasing function of *r *in the case of a Gaussian input. The demonstration of this result will directly use theorem 5.1.1.

**Proposition 5.1.1**. *V is a monotonically decreasing function in r for any monotonically decreasing synaptic weight function W*.

*Proof*. Differentiating ℳ with respect to *r *yields:

We have to compute

It is result of elementary hyperbolic trigonometry that(25)

we let *ρ *= tanh(*r*), *ρ*' = tanh(*r*') and define

It follows that

and

We conclude that if *ρ *> tanh(*ω*) then for all 0 ≤ *ρ*' ≤ tanh(*ω*) and 0 ≤ *θ *≤ 2*π*

which implies  for *r *>*ω*, since *W*' < 0.

To see that it is also negative for *r *<*ω*, we differentiate equation (24) with respect to *r*:

The following formula holds for the hypergeometric function (see Erdelyi in [32]):

It implies

Substituting in the previous equation giving  we find:

implying that:

Consequently,  for *r *<*ω*. Hence *V *is monotonically decreasing in *r *for any monotonically decreasing synaptic weight function *W*.

□

As a consequence, for our particular choice of exponential weight function (21), the radially symmetric bump is monotonically decreasing in *r*, as it will be recover in our numerical experiments in section 6.

### 5.2 Proof of theorem 5.1.1

The proof of theorem 5.1.1 goes in four steps. First we introduce some notations and recall some basic properties of the Fourier transform in the Poincaré disk. Second we prove two propositions. Third we state a technical lemma on hypergeometric functions, the proof being given in lemma F.0.4 of the appendix F. The last step is devoted to the conclusion of the proof.

#### 5.2.1 First step

In order to calculate , we use the Fourier transform in  which has already been introduced in section 4. First we rewrite  as a convolution product:

**Proposition 5.2.1**. *For all **:*(26)

*Proof*. We start with the definition of  and use the convolutional form of the integral:

In [22], Helgason proves an inversion formula for the hyperbolic Fourier transform and we apply this result to **W**:

the last equality is a direct application of lemma 4.2.1 and we can deduce that(27)

Finally we have:

which is the desired formula.   □

It appears that the study of  consists in calculating the convolution product .

**Proposition 5.2.2**. *For all z *= *k *·*O for k *∈ *G *= *SU*(1, 1) *we have:*

*Proof*. Let *z *= *k *·*O *for *k *∈ *G *we have:

for all *g*, *k *∈ *G*, *_λ_*(*g*^-1^*k *·*O*) = *_λ_*(*k*^-1^*g *·*O*) so that:

□

#### 5.2.2 Second step

In this part, we prove two results:

• the mapping  is a radial function, i.e. it depends only upon the variable *r*.

• the following equality holds for *z *= tanh(*r*)*e^iθ^*:

**Proposition 5.2.3**. *If z *= *k *·*O and z is written ** with r *= *d*_2_(*z*, *O*) *in hyperbolic polar coordinates the function ** depends only upon the variable r*.

*Proof*. If , then *z *= rot*_θ _a_r _*·*O *and *k*^-1 ^= *a*_-*r*_rot_-*θ*_. Similarly *z*' = rot_*θ' *_*a*_*r*'_·*O*. We can write thanks to the previous proposition 5.2.2:

which, as announced, is only a function of *r*.   □

We now give an explicit formula for the integral .

**Proposition 5.2.4**. *For all ** we have*:

*Proof*. We first recall a formula from [22].

**Lemma 5.2.1**. *For all g *∈ *G the following equation holds*:

*Proof*. See [22].   □

It follows immediately that for all  and *r *∈ ℝ we have:

We integrate this formula over the hyperbolic ball *B_h_*(0, *ω*) which gives:

and we exchange the order of integration:

We note that the integral  does not depend upon the variable *b *= *e^iϕ^*. Indeed:

and indeed the integral does not depend upon the variable *b*:

Finally, we can write:

because Φ_λ _= Φ_-λ _(as solutions of the same equation).

This completes the proof that:

   □

#### 5.2.3 Third step

We state a useful formula.

**Lemma 5.2.2**. *For all ω *> 0 *the following formula holds*:

*Proof*. See lemma F.0.4 of appendix F.   □

#### 5.2.4 The main result

At this point we have proved the following proposition thanks to propositions 5.2.1 and 5.2.4.

**Proposition 5.2.5**. *If *, *is given by the following formula*:

where

We are now in a position to obtain the analytic form for  of theorem 5.1.1. We prove that

Indeed, in hyperbolic polar coordinates, we have:

On the other hand:

This yields

and we use lemma (5.2.2) to establish (24).

### 5.3 Linear stability analysis

We now analyse the evolution of small time-dependent perturbations of the hyperbolic stationary-pulse solution through linear stability analysis. We use classical tools already developped in [31,29].

#### 5.3.1 Spectral analysis of the linearized operator

Equation (20) is linearized about the stationary solution *V*(*r*) by introducing the time-dependent perturbation:

This leads to the linear equation:

We separate variables by setting *ϕ*(*z*, *t*) = *ϕ*(*z*)*e^βt ^*to obtain the equation:

Introducing the hyperbolic polar coordinates  and using the result:

we obtain:

Note that we have formally differentiated the Heaviside function, which is permissible since it arises inside a convolution. One could also develop the linear stability analysis by considering perturbations of the threshold crossing points along the lines of Amari [20]. Since we are linearizing about a stationary rather than a traveling pulse, we can analyze the spectrum of the linear operator without the recourse to Evans functions.

With a slight abuse of notation we are led to study the solutions of the integral equation:(28)

where the following equality derives from the definition of the hyperbolic distance in equation (25):

**Essential spectrum **If the function *ϕ *satisfies the condition

then equation (28) reduces to:

yielding the eigenvalue:

This part of the essential spectrum is negative and does not cause instability.

**Discrete spectrum **If we are not in the previous case we have to study the solutions of the integral equation (28).

This equation shows that *ϕ*(*r*, *θ*) is completely determined by its values *ϕ*(*ω*, *θ*) on the circle of equation *r *= *ω*. Hence, we need only to consider *r *= *ω*, yielding the integral equation:

The solutions of this equation are exponential functions *e^γθ^*, where *γ *satisfies:

By the requirement that *ϕ *is 2*π*-periodic in *θ*, it follows that *γ *= *in*, where *n *∈ ℤ. Thus the integral operator with kernel  has a discrete spectrum given by:

*β_n _*is real since:

Hence,

We can state the following proposition:

**Proposition 5.3.1**. *Provided that for all n *≥ 0, *β_n _*< 0 *then the hyperbolic stationary pulse is stable*.

We now derive a reduced condition linking the parameters for the stability of hyperbolic stationary pulse.

**Reduced condition **Since  is a positive function of *r*, it follows that:

Stability of the hyperbolic stationary pulse requires that for all *n *≥ 0, *β_n _*< 0. This can be rewritten as:

Using the fact that *β_n _*≤ *β*_0 _for all *n *≥ 1, we obtain the reduced stability condition:

Where

From (22) we have:

Where

We have previously established that  and *I'*(*ω*) is negative by definition. Hence, letting  we have

By substitution we obtain another form of the reduced stability condition:(29)

We also have:

and

showing that the stability condition (29) is satisfied when *N'*(*ω*) > 0 and is not satisfied when *N'*(*ω*) > 0.

**Proposition 5.3.2 **(Reduced condition). If *N'*(*ω*) > 0 *then for all n *≥ 0, *β_n _*< 0 *and the hyperbolic stationary pulse is stable*.

## 6 **Numerical results**

The aim of this section is to numerically solve (13) for different values of the parameters. This implies developing a numerical scheme that approaches the solution of our equation, and proving that this scheme effectively converges to the solution.

Since equation (13) is defined on , computing the solutions on the whole hyperbolic disk has the same complexity as computing the solutions of usual Euclidean neural field equations defined on ℝ^2^. As most authors in the Euclidean case [31,27,26,29], we reduce the domain of integration to a compact region of the hyperbolic disk. Practically, we work in the Euclidean ball of radius *a *= 0.5 and center 0. Note that a Euclidean ball centered at the origin is also a centered hyperbolic ball, their radii being different.

We have divided this section into four parts. The first part is dedicated to the study of the discretization scheme of equation (13). In the following two parts, we study the solutions for different connectivity functions: an exponential function, section 6.2, and a difference of Gaussians, section 6.3.

### 6.1 Numerical schemes

Let us consider the modified equation of (13):(30)

We assume that the connectivity function satisfies the conditions (**C1**)-(**C2**). Moreover we express *z *in (Euclidean) polar coordinates such that  and . The integral in equation (30) is then:

We define  to be the rectangle .

### 6.1.1 Discretization scheme

We discretize  in order to turn (30) into a finite number of equations. For this purpose we introduce  and ,

and obtain the (*N *+ 1) (*M *+1) equations:

which define the discretization of (30):(31)

where . Similar definitions apply  and . Moreover:

 is the space of the matrices of size *n *× *p *with real coefficients. It remains to discretize the integral term. For this as in [33], we use the rectangular rule for the quadrature so that for all  we have:

We end up with the following numerical scheme, where  (resp. ) is an approximation of  (resp. ), :

With 

### 6.1.2 Discussion

We discuss the error induced by the rectangular rule for the quadrature. Let *f *be a function which is  on a rectangular domain [*a*, *b*] × [*c*, *d*]. If we denote by *E*_*f *_this error, then  where *m *and *n *are the number of subintervals used and  where, as usual, *α *is a multi-index. As a consequence, if we want to control the error, we have to impose that the solution is, at least,  in space.

Four our numerical experiments we use the specific function ode45 of Matlab which is based on an explicit Runge-Kutta (4,5) formula (see [34] for more details on Runge-Kutta methods).

We can also establish a proof of the convergence of the numerical scheme which is exactly the same as in [33] excepted that we use the theorem of continuous dependence of the solution for ordinary differential equations.

### 6.2 Purely excitatory exponential connectivity function

In this subsection, we give some numerical solutions of (13) in the case where the connectivity function is an exponential function, , with *b *a positive parameter. Only excitation is present in this case. In all the experiments we set *α *= 0.1 and  with *μ *= 10.

**Constant input **We fix the external input *I*(*z*) to be of the form:

In all experiments we set  and *σ *= 0.05, this means that the input has a sharp profile centered at 0.

**Figure 2:**
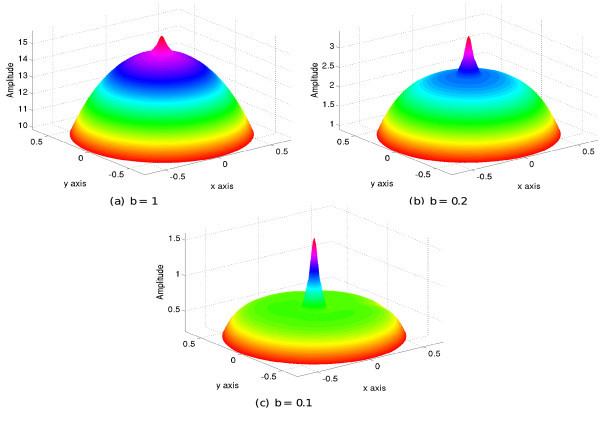
**Plots of the solution of equation (13) at *T *= 2500 for the values *μ *= 10, *α *= 0.1 and for decreasing values of the width *b *of the connectivity, see text**.

We show in Figure 2 plots of the solution at time *T *= 2500 for three different values of the width *b *of the exponential function. When *b *= 1, the whole network is highly excited, whereas as *b *changes from 1 to 0.1 the amplitude of the solution decreases, and the area of high excitation becomes concentrated around the external input.

**Figure 3:**
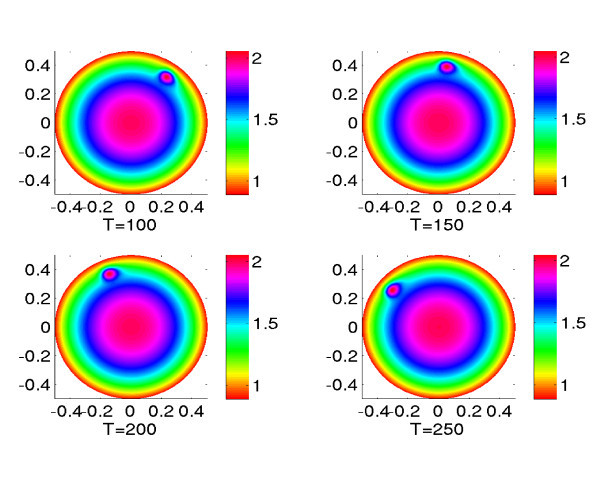
**Plots of the solution of equation (13) in the case of an exponential connectivity function with *b *= 0.1 at different times with a time-dependent input, see text**.

**Variable input **In this paragraph, we allow the external current to depend upon the time variable. We have:

where . This is a bump rotating with angular velocity Ω_0 _around the circle of radius *r*_0 _centered at the origin. In our numerical experiments we set *r*_0 _= 0.4, Ω_0 _= 0.01,  and *σ *= 0.05. We plot in Figure 3 the solution at different times *T *= 100, 150, 200, 250.

**Figure 4:**
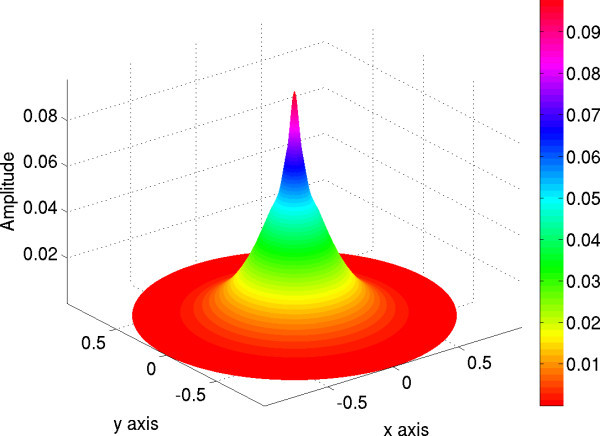
**Plot of a bump solution of equation (22) for the values *α *= 1, *κ *= 0.04, *ω *= 0.18 and for *b *= 0.2 for the width of the connectivity, see text**.

**High gain limit **We consider the high gain limit *μ *→ ∞ of the sigmoid function and we propose to illustrate section 5 with a numerical simulation. We set *α *= 1, *κ *= 0.04, *ω *= 0.18. We fix the input to be of the form:

with  and *σ *= 0.05. Then the condition of existence of a stationary pulse (23) is satisfied, see Figure 1. We plot a bump solution according to (23) in Figure 4.

### 6.3 Excitatory and inhibitory connectivity function

We give some numerical solutions of (13) in the case where the connectivity function is a difference of Gaussians, which features an excitatory center and an inhibitory surround:

We illustrate the behaviour of the solutions when increasing the slope *μ *of the sigmoid. We set the  so that it is equal to 0 at the origin and we choose the external input equal to zero, *I*(*z*, *t*) = 0. In this case the constant function equal to 0 is a solution of (13).

**Figure 5:**
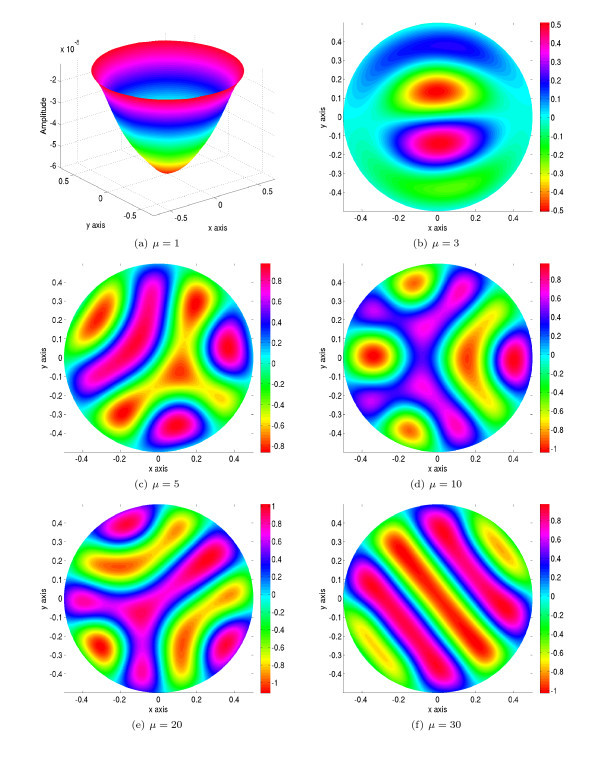
**Plots of the solutions of equation (13) in the case where the connectivity function is the difference of two Gaussians at time *T *= 2500 for *α *= 0.1 and for increasing values of the slope *μ *of the sigmoid, see text**.

For small values of the slope *μ*, the dynamics of the solution is trivial: every solution asymptotically converges to the null solution, as shown in top left hand corner of Figure 5 with *μ *= 1. When increasing *μ*, the stability bound, found in subsection 4.5 is no longer satisfied and the null solution may no longer be stable. In effect this solution may bifurcate to other, more interesting solutions. We plot in Figure 5, some solutions at *T *= 2500 for different values of *μ *(*μ *= 3, 5, 10, 20 and 30). We can see exotic patterns which feature some interesting symmetries. The formal study of these bifurcated solutions is left for future work.

## 7 Conclusion

In this paper, we have studied the existence and uniqueness of a solution of the evolution equation for a smooth neural mass model called the structure tensor model. This model is an approach to the representation and processing of textures and edges in the visual area V1 which contains as a special case the well-known ring model of orientations (see [1,2,19]). We have also given a rigorous functional framework for the study and computation of the stationary solutions to this nonlinear integro-differential equation. This work sets the basis for further studies beyond the spatially periodic case studied in [15], where the hypothesis of spatial periodicity allows one to replace the unbounded (hyperbolic) domain by a compact one, hence making the functional analysis much simpler.

We have completed our study by constructing and analyzing spatially localised bumps in the high-gain limit of the sigmoid function. It is true that networks with Heaviside nonlinearities are not very realistic from the neurobiological perspective and lead to difficult mathematical considerations. However, taking the high-gain limit is instructive since it allows the explicit construction of stationary solutions which is impossible with sigmoidal nonlinearities. We have constructed what we called a hyperbolic radially symmetric stationary-pulse and presented a linear stability analysis adapted from [31]. The study of stationary solutions is very important as it conveys information for models of V1 that is likely to be biologically relevant. Moreover our study has to be thought of as the analog in the case of the structure tensor model to the analysis of tuning curves of the ring model of orientations (see [1,2,19,35]). However, these solutions may be destabilized by adding lateral spatial connections in a spatially organized network of structure tensor models; this remains an area of future investigation. As far as we know, only Bressloff and coworkers looked at this problem (see [3,11-14,4]).

Finally, we illustrated our theoretical results with numerical simulations based on rigorously defined numerical schemes. We hope that our numerical experiments will lead to new and exciting investigations such as a thorough study of the bifurcations of the solutions of our equations with respect to such parameters as the slope of the sigmoid and the width of the connectivity function.

## Competing interests

The authors declare that they have no competing interests.

## A Isometries of 

We briefly descrbies the isometries of , i.e the transformations that preserve the distance *d*_2_. We refer to the classical textbooks in hyperbolic goemetry for details, e.g, [17]. The direct isometries (preserving the orientation) in  are the elements of the special unitary group, noted SU(1, 1), of 2 × 2 Hermitian matrices with determinant equal to 1. Given:

an element of SU(1, 1), the corresponding isometry *γ *in  is defined by:(32)

Orientation reversing isometries of  are obtained by composing any transformation (32) with the reflection . The full symmetry group of the Poincaré disc is therefore:

Let us now describe the different kinds of direct isometries acting in . We first define the following one parameter subgroups of SU(1, 1):

Note that  and also *a_r_*·*O *= tanh *r*, with *O *being the center of the Poincaré disk that is the point represented by *z *= 0.

The group *K *is the orthogonal group O(2). Its orbits are concentric circles. It is possible to express each point  in hyperbolic polar coordinates:  and *r *= *d*_2_(*z*, 0).

The orbits of *A *converge to the same limit points of the unit circle , *b*_±1 _= ±1 when *r *→ ±∞. They are circular arcs in  going through the points *b*_1 _and *b*_-1_.

The orbits of *N *are the circles inside  and tangent to the unit circle at *b*_1_. These circles are called *horocycles *with base point *b*_1_. *N *is called the horocyclic group. It is also possible to express each point  in horocyclic coordinates: *z_s_a_r_*·*O*, where *n_s _*are the transformations associated with the group *N *(*s *∈ ℝ) and *a_r _*the transformations associated with the subroup *A *(*r *∈ ℝ).

**Iwasawa decomposition **The following decomposition holds, see [36]:

This theorem allows us to decompose any isometry of  as the product of at most three elements in the groups, *K*, *A *and *N*.

## B Volume element in structure tensor space

Let  be a structure tensor

Δ^2 ^its determinant, Δ ≥ 0.  can be written

Where  has determinant 1. Let *z *= *z*_1 _+ *iz*_2 _be the complex number representation of  in the Poincaré disk . In this part of the appendix, we present a simple form for the volume element in full structure tensor space, when parametrized as (Δ, *z*).

**Proposition B.0.1**. *The volume element in *(Δ, *z*_1_, *z*_2_) *coordinates is*(33)

*Proof*. In order to compute the volume element in (Δ, *z*_1_, *z*_2_) space, we need to express the metric  in these coordinates. This is obtained from the inner product in the tangent space  at point  of SDP(2). The tangent space is the set S(2) of symmetric matrices and the inner product is defined by:

We note that . We write *g *instead of . A basis of  (or  for that matter) is given by:

and the metric is given by:

The determinant  of  is equal to *G*/Δ^6^, where *G *is the determinant of . *G *is found to be equal to 2. The volume element is thus:

We then use the relations:

where , *i *= 1, 2, 3 is given by:

The determinant of the Jacobian of the transformation (*x*_1_, *x*_2_, *x*_3_) → (Δ, *z*_1_, *z*_2_) is found to be equal to:

Hence, the volume element in (Δ, *z*_1_, *z*_2_) coordinates is

   □

## C Global existence of solutions

**Theorem C.0.1**. *Let  be an open connected set of a real Banach space  and J be an open interval of *ℝ. *We consider the initial value problem:*(34)

*We suppose that  and is locally Lipschitz with respect to its second argument. Then for all , there exists τ *> 0 *and  unique solution of (34)*.

**Lemma C.0.1**. *Under hypotheses of theorem C.0.1, if  and  are two solutions and if there exists t*_0 _∈ *J*_1 _∩ *J*_2 _*such that ***V**_1_(*t*_0_) *= ***V**_2_(*t*_0_) *then:*

This lemma shows the existence of a larger interval *J*_0 _on which the initial value problem (34) has a unique solution. This solution is called the maximal solution.

**Theorem C.0.2**. *Under hypotheses of theorem C.0.1, let ** be a maximal solution. We denote by b the upper bound of J and β the upper bound of J*_0_*. Then either β = b or for all compact set , there exists η < β such that:*

*We have the same result with the lower bounds*.

**Theorem C.0.3**. *We suppose  and is globally Lipschitz with respect to its second argument. Then for all , there exists a unique  solution of *(34).

## D Proof of lemma 3.1.1

**Lemma D.0.2**. *When W is only a function of , then  does not depend upon the variable *.

*Proof. We work in *(*z*, Δ) coordinates and we begin by rewriting the double integral (6) for all :

The change of variable  yields:

And it establishes that  does not depend upon the variable Δ. To finish the proof, we show that the following integral does not depend upon the variable :(35)

where *f *is a real-valued function such that Ξ(*z*) is well defined.

We express *z *in horocyclic coordinates: *z_s_a_r_*.*O *(see appendix A) and (35) becomes:

With the change of variable *s *− *s' *= −*xe*^2*r*^, this becomes:

The relation  (proved e.g. in [22]) yields:

with  and , which shows that Ξ(*z*) does not depend upon the variable *z*, as announced.   □

## E Proof of lemma 3.1.2

In this section we prove the following lemma.

**Lemma E.0.3**. *When W is the following Mexican hat function:*

where:

*with *0 ≤ *σ*_1 _≤ *σ*_2 _*and *0 ≤ *A *≤ 1.

Then:

where **erf **is the error function defined as:

*Proof*. We consider the following double integrals:(36)

so that:

Since the variables are separable, we have:

One can easily see that:

We now give a simplified expression for Ξ*_i_*. We set  and then we have, because of lemma 3.1.1:

The change of variable *x *= arctanh(*r*) implies  and yields:

then we have a simplified expression for Ξ*_i_*:

   □

## F Proof of lemma 5.2.2

**Lemma F.0.4**. *For all ω *> 0 *the following formula holds:*

*Proof*. We write *z *in hyperbolic polar coordinates, *z *= tanh (*r*) *e^iθ ^*(see appendix A). We have:

Because of the above definition of Φ_λ_, this reduces to

In [22] Helgason proved that:

with . We then use the formula obtained by Erdelyi in [32]:

Using some simple hyperbolic trigonometry formulae we obtain:

from which we deduce

Finally we use the equality shown in [32]:

In our case we have: *a = ν*, *b = *1 - *ν*, *c *= 2 and *z *= - sinh (*ω*)^2^, so . We obtain

Since Hypergometric functions are symmetric with respect to the first two variables:

we write

which yields the announced formula

□
